# Intent to Receive Pandemic Influenza A (H1N1) Vaccine, Compliance with Social Distancing and Sources of Information in NC, 2009

**DOI:** 10.1371/journal.pone.0011226

**Published:** 2010-06-18

**Authors:** Jennifer A. Horney, Zack Moore, Meredith Davis, Pia D. M. MacDonald

**Affiliations:** 1 Department of Epidemiology, University of North Carolina Gillings School of Global Public Health, Chapel Hill, North Carolina, United States of America; 2 North Carolina Division of Public Health, Raleigh, North Carolina, United States of America; 3 North Carolina Institute for Public Health, Chapel Hill, North Carolina, United States of America; HelmholtzZentrum München, Germany

## Abstract

**Background:**

Public adherence to influenza vaccination recommendations has been low, particularly among younger adults and children under 2, despite the availability of safe and effective seasonal vaccine. Intention to receive 2009 pandemic influenza A (H1N1) vaccine has been estimated to be 50% in select populations. This report measures knowledge of and intention to receive pandemic vaccine in a population-based setting, including target groups for seasonal and H1N1 influenza.

**Methodology and Principal Findings:**

On August 28–29, 2009, we conducted a population-based survey in 2 counties in North Carolina. The survey used the 30×7 two-stage cluster sampling methodology to identify 210 target households. Prevalence ratios (PR) and 95% confidence intervals (CI) were estimated. Knowledge of pandemic influenza A (H1N1) vaccine was high, with 165 (80%) aware that a vaccine was being prepared. A total of 133 (64%) respondents intended to receive pandemic vaccine, 134 (64%) intended to receive seasonal vaccine, and 109 (53%) intended to receive both. Reporting great concern about H1N1 infection (PR 1.55; 95%CI: 1.30, 1.85), receiving seasonal influenza vaccine in 2008–09 (PR 1.47; 95%CI: 1.18, 1.82), and intending to receive seasonal influenza vaccine in 2009–10 (PR 1.27; 95%CI: 1.14, 1.42) were associated with intention to receive pandemic vaccine. Not associated were knowledge of vaccine, employment, having children under age 18, gender, race/ethnicity and age. Reasons cited for not intending to get vaccinated include not being at risk for infection, concerns about vaccine side effects and belief that illness caused by pandemic H1N1 would be mild. Forty-five percent of households with children under 18 and 65% of working adults reported ability to comply with self-isolation at home for 7–10 days if recommended by authorities.

**Conclusions and Significance:**

This is the first report of a population based rapid assessment used to assess knowledge and intent to receive pandemic vaccine in a community sample. Intention to receive pandemic and seasonal vaccines was higher than previously published reports. To reach persons not intending to receive pandemic vaccine, public health communications should focus on the perceived risk of infection and concerns about vaccine safety.

## Introduction

On April 26, 2009, the Acting Secretary of the US Department of Health and Human Services declared a public health emergency related to the emergence and spread of a novel influenza A (H1N1) virus initially identified in California and Texas. North Carolina reported its first confirmed case of infection with the 2009 pandemic influenza A (H1N1) virus (pandemic H1N1) on May 3, 2009. By June, the World Health Organization had declared a phase 6 pandemic, indicating global spread. North Carolina continued to report widespread influenza activity into the fall of 2009.

Vaccination is considered to be an effective method for preventing influenza and influenza-related complications. Soon after the pandemic H1N1 virus was identified, it was recognized that the 2009–2010 seasonal vaccine was not likely to be protective against infection with the novel virus [1]. Pandemic H1N1 monovalent vaccine, produced by the same manufacturers and in the same way as the seasonal vaccine, was made available nationally beginning in October 2009. Two presentations of new vaccine were produced in the United States; a nasal spray live attenuated influenza vaccine and an inactivated vaccine administered by intramuscular injection [2].

The U.S. Centers for Disease Control and Prevention (CDC) have released recommendations for the use of pandemic H1N1 vaccine in the United States [3]. The five initial target groups are estimated to include 159 million persons who should be the first to receive vaccine. These include pregnant women, persons who live with or provide care for infants aged <6 months, health-care and emergency medical services personnel, children and young adults aged 6 months–24 years, and persons aged 25–64 years who have medical conditions that put them at higher risk for influenza-related complications. Additional groups should be vaccinated as supply allows.

In the United States, public adherence to influenza vaccination recommendations has been low in recent years despite the availability of safe and effective seasonal influenza vaccine. While 83% of the United States population is included in one or more of the target groups for whom seasonal influenza vaccination is recommended, only 33% received an influenza vaccination during the 2008–09 influenza season [4]. Few young adults are targeted for seasonal vaccine, and uptake among healthy persons 18 years to 49 years in 2008 was poor at 20% [5].

Several studies have examined the intention to receive pandemic H1N1 vaccine. In an internet panel survey conducted by RAND Corporation (www.rand.org) in May and June 2009, 49.6% of adults indicated intention to receive the pandemic H1N1 vaccine [6]. Intention to receive pandemic H1N1 vaccine was strongly associated with intention to receive seasonal vaccine; those who indicated they would receive seasonal vaccine in 2009–10 were twice as likely to report intention to receive pandemic H1N1 vaccine.

A telephone survey conducted by Harvard University researchers in September 2009 concluded that 40% of adults were absolutely certain they would receive the pandemic H1N1 vaccine and 51% of parents were absolutely certain they would vaccinate their children [7]. A San Francisco-based poll of registered voters reported that 72% of respondents would be likely to get the vaccine if their physician or public health officials recommended it, although only 51% of respondents were very worried about H1N1. Women and Latinos included in the poll expressed higher levels of concern than men and respondents of other races/ethnicities [8].

To mount an effective public health response, officials in North Carolina needed to know more about the intent of North Carolinians, particularly those in target groups, to receive the pandemic H1N1 vaccine before the vaccine was made available for distribution. In addition to understanding more about the population's intent to be vaccinated for seasonal influenza, officials also needed to know more about residents' knowledge and concerns about pandemic H1N1 vaccine, sources of health information, and likelihood of following self-isolation recommendations.

The North Carolina Center for Public Health Preparedness and the North Carolina Preparedness and Emergency Response Research Center at the University of North Carolina Gillings School of Global Public Health partnered with the North Carolina Division of Public Health to conduct a population-based survey in 2 North Carolina counties. The purpose of the survey was to determine the intent of the population to receive the pandemic H1N1 and seasonal influenza vaccines in fall 2009, ability to comply with recommended isolation and social distancing measures, and the sources by which residents received health information about pandemic influenza A (H1N1).

## Methods

Two counties in North Carolina were selected for inclusion in the study, based on proximity to the University of North Carolina at Chapel Hill and diversity of their populations. Both counties are approximately 80% white, non-Hispanic and have similar population density. Orange County, home to the University, has a high median household income, high home values, and a high percentage of the population with at least a bachelor's degree when compared to North Carolina averages. Alamance County has a higher percentage of African-American residents than Orange County and a higher percentage of Hispanic residents than Orange County or the state as a whole.

In order to quickly collect actionable data, a rapid needs assessment was conducted using the CDC-validated 30×7 two-stage cluster sampling methodology to identify 210 target households [9]. This random household cluster sampling scheme has been validated and used effectively for rapid assessment and estimation of a variety of population-level public health needs. Maps of the 2 counties that delineate each lot by land ownership were obtained from the respective county governments, and 30 census block groups (the second smallest geographic subdivision for which the U.S. Census Bureau tabulates sample data) in the 2 counties were randomly selected based on probability proportionate to population size sampling. Within each selected block group, 7 interview locations were chosen from a simple random sample of all existing parcels using a geographic information systems-based (GIS) survey site selection toolkit developed by the North Carolina Division of Public Health in ESRI ArcMap 9.2 (Redlands, CA).

Surveys were implemented on Friday, August 28 and Saturday, August 29, 2009 via in-person interviews with 1 adult member of each selected household. Interviews were conducted between 10 a.m.–6 p.m. Data were electronically recorded during the interview using global positioning systems-equipped Trimble Recon field data collectors. Two-person interviewer teams, including student volunteers from the UNC Gillings School of Global Public Health's Team Epi-Aid program and health department staff members, were routed to each location with a map generated with ESRI ArcPad 6.0.3 Street Map USA (Redlands, CA). Interview teams assigned to census blocks with large Hispanic populations included a Spanish speaker, and a translated survey instrument was available. Oral informed consent was obtained from each respondent. This research received approval by the Institutional Review Board of the University of North Carolina at Chapel Hill Gillings School of Global Public Health (Public Health IRB #09-1518).

The survey included 26 questions and took 15 minutes to administer (Appendix S1). Intention to receive seasonal and pandemic H1N1 vaccine was measured by asking respondents to report whether or not they intended to get vaccinated when each vaccine was available. For each vaccine, if respondents indicated that they intended to get vaccinated, interviewers read a list of reasons and asked respondents to indicate if each reason applied to them. A similar procedure was followed for those who reported that they did not intend to be vaccinated. Those intending to be vaccinated were asked in what venue they would receive the vaccination. Knowledge of pandemic influenza vaccination was assessed by asking respondents if they were aware that an H1N1 (swine flu) vaccine was being prepared and if they were aware that 2 separate doses of this vaccine might be recommended. Concern about pandemic H1N1 was measured by asking respondents to report whether they were very concerned, somewhat concerned, or not at all concerned about H1N1 infection.

Next, respondents were asked about conditions that could facilitate or hinder compliance with recommended isolation and social distancing measures recommended by the North Carolina Division of Public Health. First, respondents reported their employment status and whether or not they had children under the age of 18 living at home. Those reporting full- or part-time employment were asked whether or not they had paid sick leave. All respondents were asked the number of days they would be able to stay home, away from people who were not a part of their household, if they were infected with H1N1 and self-isolation was recommended by health authorities. Those with children under 18 years of age were asked to categorize the age of the children (<6 months, 6 months to 4 years, 5 years to 17 years) and to indicate if their children attended school or day care and their intention to vaccinate their children against H1N1. Respondents with children were asked to report the number of days they would be able to keep their child(ren) at home, away from people who were not a part of their household, if their child was infected with H1N1.

Interviewers read a list of potential sources of information about pandemic H1N1 to respondents, who indicated whether they had received any information from each. Limited demographic data, including gender, age, race, and ethnicity, were also collected.

Bivariate analyses were performed using generalized linear models to identify associations between intention to receive pandemic H1N1 vaccine and demographic, knowledge, and behavioral variables. Prevalence ratios (PR) and 95% confidence intervals (CI) were estimated. All statistical analyses were conducted in SAS 9.1.3 (Cary, NC).

## Results

Of 258 households visited in the population-based survey, individuals from 207 households completed the survey (response rate = 80%). The median age of respondents was 49 years (range 18–92 years), 56% were female (n = 115), and the majority were white, non-Hispanic (n = 140).

A total of 133 (64%) respondents reported that they intended to receive pandemic H1N1 vaccine (Table 1) and 109 (53%) intended to receive both pandemic H1N1 and seasonal influenza vaccines [10]. Knowledge of pandemic H1N1 vaccine, age, gender, race/ethnicity, work status, and having children under age 18 living at home were not significantly associated (p<0.05) with the respondent's intention to receive pandemic H1N1 vaccine. Those who reported receiving the seasonal influenza vaccine in 2008–09 were 1.47 (95%CI: 1.18, 1.82) times as likely to report intention to receive pandemic H1N1 vaccine. Respondents who reported that they intended to receive seasonal influenza vaccine in 2009–10 were 1.27 (95%CI: 1.14, 1.42) times as likely to report intention to receive pandemic H1N1 vaccine. Those who reported that they were very concerned about being infected with pandemic H1N1 were 1.55 (95%CI: 1.30, 1.85) times as likely to report intention to receive pandemic H1N1 vaccine compared with those who were not at all or only somewhat concerned with infection.

**Table 1:** Factors potentially associated with intent to receive pandemic H1N1 vaccination, North Carolina, 2009 (N = 207).

Variable Description	Intend to Receive Pandemic Vaccine (n = 133)		Do Not Intend to Receive Pandemic Vaccine (n = 74)		Prevalence Ratio (95% CI)
	n	%	n	%	
Knowledge of Pandemic Vaccine					
No/Don't Know	30	71.43	12	28.57	REF
Yes	103	62.42	62	37.58	1.09 (0.95, 1.25)_
Level of Concern					
Not at all Concerned	26	45.61	31	54.39	REF
Somewhat Concerned	57	61.96	35	38.04	0.94 (0.76, 1.15)
Very Concerned	50	86.21	8	13.79	1.55 (1.30, 1.85)
Received Seasonal Flu Vaccine in 2008–09					
No	53	51.96	49	48.04	REF
Yes	80	76.19	25	23.81	1.47 (1.18, 1.82)
Intend to Receive Seasonal Flu Vaccine in 2009–10					
No	24	32.00	51	68.00	REF
Yes	109	76.76	23	23.24	1.27 (1.14, 1.42)
Employment Status					
Homemaker, Student, Retired, Disabled, Unemployed	75	68.81	34	31.19	REF
Work Full- or Part-Time	58	59.18	40	40.82	0.86 (0.70, 1.06)
Children in Home					
No	85	63.91	48	36.09	REF
Yes	48	64.86	26	35.14	0.96 (0.80, 1.22)
Gender					
Female	71	61.74	44	38.26	REF
Male	61	67.03	30	32.97	1.07 (0.87, 1.31)
Missing	1	100.00	0	0.00	
Race/Ethnicity					
White, Non-Hispanic	87	62.14	53	37.86	REF
Other	46	69.70	20	30.30	1.12 (0.91, 1.38)
Missing	0	0.00	1	100.00	
Age					
18–24	14	61.54	8	38.46	REF
25–64	88	63.77	50	36.23	0.96 (0.78, 1.19)
65 and over	31	65.96	16	34.04	1.03 (0.81, 1.30)

CI = Confidence interval.

The association between seasonal influenza vaccination and intention to receive pandemic H1N1 vaccine was only significant among those 25–64 years of age. Respondents in that age group who reported receiving the seasonal influenza vaccine in 2008–09 were 1.54 (95% CI: 1.19, 1.98) times as likely to report intention to receive pandemic H1N1 vaccine while those between 18 and 24 and those over age 65 were not significantly more likely to report intention to receive H1N1 based on receiving seasonal influenza vaccine in the prior year. When considering intention to receive seasonal vaccine this year, those age 25–64 intending to receive seasonal vaccine in 2009–10 were 1.33 (95% CI: 1.17, 1.51) times as likely to report intention to receive pandemic H1N1 vaccine while those between 18 and 24 and those over age 65 were not significantly more likely to report intention to receive H1N1 based on intention to receive seasonal influenza vaccine in the current year. Among those who reported that they were very concerned about being infected with pandemic H1N1, those age 25–64 were 1.50 (95% CI: 1.20, 1.88) times as likely to report intention to receive pandemic H1N1 vaccine while those over age 65 were 1.56 (95% CI: 1.12, 2.18) times as likely to report intention to receive pandemic H1N1 vaccine.

A total of 132 (64%) respondents reported that they intended to receive seasonal influenza vaccine (Table 2). Those who reported receiving seasonal vaccine during the 2008–09 influenza season were 3.04 (95%CI: 2.27, 4.06) times as likely to report intention to receive seasonal vaccine in 2009–10. When stratified by age group, the association between past seasonal influenza vaccination and intention to receive seasonal vaccine was only significant among those 25–64, who were 3.34 (95% CI: 2.34, 4.77) times as likely to report intention to receive seasonal vaccine based on seasonal vaccine receipt in the previous year. Those who reported that they intended to receive pandemic H1N1 vaccine in 2009–10 were 2.64 (95%CI: 1.86, 3.74) times as likely to report that they intended to receive seasonal vaccine this year. When stratified by age group, the association between intention to receive pandemic H1N1 vaccine and intention to receive seasonal vaccine was only significant among those 25–64, who were 2.76 (95% CI: 1.74, 4.37) times as likely to report intention to receive seasonal vaccine based on intention to receive pandemic H1N1 vaccine. Level of concern about contracting pandemic H1N1 was also significantly associated with intention to receive 2009–10 seasonal vaccine (PR = 1.24; 95% CI: 1.02, 1.52). Knowledge of pandemic H1N1 vaccine, gender, race/ethnicity, and having children under age 18 living at home were not significantly associated (p<0.05) with the respondent's intention to receive seasonal vaccine.

**Table 2:** Factors potentially associated with intention to receive vaccination for seasonal influenza, North Carolina, 2009 (N = 207).

Variable Description	Intend to Receive Seasonal Vaccine (n = 132)		Do Not Intend to Receive Seasonal Vaccine (n = 75)		Prevalence Ratio (95% CI)
	n	%	n	%	
Knowledge of Pandemic Vaccine					
No/Don't Know	27	64.29	15	35.71	REF
Yes	105	63.63	60	36.37	1.00 (0.85, 1.18)
Level of Concern					
Not at all Concerned	28	49.12	29	50.88	REF
Somewhat Concerned	61	66.30	31	33.70	1.07 (0.88, 1.32)
Very Concerned	43	74.14	15	25.86	1.24 (1.02, 1.52)
Received Seasonal Flu Vaccine in 2008–09					
No	32	31.37	70	68.63	REF
Yes	100	95.24	5	4.76	3.04 (2.27, 4.06)
Intend to Receive Pandemic Vaccine in 2009–10					
No	23	31.08	51	68.92	REF
Yes	109	81.95	24	18.05	2.64 (1.86, 3.74)
Employment Status					
Homemaker, Student, Retired, Disabled, Unemployed	79	72.48	30	27.52	REF
Work Full-or Part-Time	53	54.08	45	45.92	0.75 (0.60, 0.93)
Children in Home					
No	86	64.66	47	35.34	REF
Yes	46	62.16	28	37.84	1.04 (0.84, 1.29)
Gender					
Female	73	63.48	42	36.52	REF
Male	58	63.74	33	36.26	0.99 (0.80, 1.22)
Missing	1	100.00	0	0.00	
Race/Ethnicity					
White, Non-Hispanic	93	66.43	47	33.57	REF
Other	39	59.09	27	40.91	0.91 (0.72, 1.15)
Missing	0	0.00	1	100.00	
Age					
18–24	12	54.55	10	45.45	REF
25–64	82	59.42	56	40.58	0.81 (0.66, 0.98)
65 and over	39	82.98	8	17.02	1.42 (1.18, 1.71)
Missing	0	0.00	1	100.00	

CI = Confidence interval.

Those who reported working either part- or full-time were only 0.75 (95%CI: 0.60, 0.93) times as likely to report intention to receive the seasonal vaccine in the 2009–10 influenza season as compared to those who were homemakers, students, disabled, retired or unemployed. Importantly, those age 65 and over were 1.42 (95%CI: 1.18, 1.71) times as likely as adults between 18–24 years to report intention to receive seasonal vaccine, consistent with public health recommendations for seasonal vaccine that include all adults over age 65 [11]. Respondents between the ages of 25 and 64 were 0.81 (95% CI: 0.66, 0.98) times as likely to report intention to receive seasonal vaccine in 2009–10 compared to those 18–24 years of age.

Most respondents (n = 165; 80%) were aware prior to the interview that a pandemic H1N1 vaccine was in production. However, the proportion who intended to receive pandemic H1N1 vaccine was similar among those who were and were not aware that the vaccine was in production. While only 75 (36%) respondents were aware the pandemic H1N1 vaccine may require 2 doses, this awareness was not associated with intent to receive the vaccine. More than two-thirds of respondents (n = 132; 64%) reported they knew where they intended to get the H1N1 vaccine, with most (n = 62) planning to get the vaccine at the physician's office. Twenty-one reported planning to get the vaccine at their local health department, while 20 planned to get vaccinated at their place of employment. Sixty percent (n = 125) of those surveyed reported having private health insurance, while 26% (n = 53) were covered by Medicare or Medicaid and 13% (n = 27) were uninsured. Neither the location where the respondent intended to get the pandemic H1N1 vaccine nor their insurance status was significantly associated with intention to receive the pandemic H1N1 vaccine.

When asked to report whether each of a list of reasons for not receiving the pandemic H1N1 vaccine applied to them, the most common reason given for not intending to receive seasonal vaccine was the belief that they would not be infected (16%).

Seventy-four (36%) of households reported a child or children under age 18 living at home including 5 infants less than 6 months old, 30 children between 6 months and 4 years and 59 children between 5 and 18 years old. Sixty-three (85%) households with children reported that their children attended day care or school. Of those with children less than 18 years old living at home, 48 (65%) households reported that they intended to vaccinate their child or children with the H1N1 vaccine. Importantly, of the 5 respondents who reported a child at home less than 6 months of age, 4 (80%) reported that they intended to vaccinate these infants, who are not eligible to receive the vaccine. Respondents reported intention to vaccinate 22 (73%) children between 6 months and 4 years of age and 36 (61%) children between 5 and 18 years of age with the H1N1 vaccine. Forty-five (61%) households expressed intent to vaccinate their children with both vaccines, including 63% among households with children between 6 months and 4 years of age and 46% among households with children between 5 and 18 years of age. Thirty-three (45%) of those with children under 18 in their household reported being able to keep their children at home and away from people who were not a part of their household for 7–10 days or as long as necessary.

Forty-seven percent (n = 98) of respondents reported being employed either full-time or part-time. Of those who reported full- or part-time employment, 41% (n = 40) did not have access to paid sick leave. However, 65% (n = 64) of those who were employed full- or part-time reported that they could stay at home and away from people who were not a part of their household for 7–10 days or as long as necessary.

## Discussion

This study found some similarities and differences regarding intent to receive vaccination for pandemic H1N1 in comparison to previous studies. Consistent with previous reports, intent to comply with recommendations for pandemic H1N1 vaccine was associated with past history of and intent to receive seasonal influenza vaccine [12]. Current knowledge of and intention to receive both the 2009–2010 pandemic H1N1 and seasonal vaccines were generally higher than those reported in other studies. This is most likely due to the widespread media coverage of the H1N1 pandemic and the vaccine since the other studies were conducted in spring of 2009 and the fact that previously published studies relied on telephone- and internet-based survey rather than face-to-face interviews, which have been demonstrated to increase social desirability bias with regard to reported adherence to health guidelines and other health behaviors [13].

The percentage of those who intended to get vaccinated in this population-based survey are higher for both vaccines than the uptake estimated for the seasonal vaccine in 2006, which ranged from 15% in non-target and 32% in target persons over 6 months of age [14]. Notably, the intent to receive pandemic H1N1 vaccine in adults over age 65, who were not included in the initial target groups for this vaccine, is high (66%) in this study, and similar to the probability of being vaccinated against H1N1 (63%) reported by Maurer [15]. However, the estimated uptake rate for seasonal vaccine among those age 65 or over in this study was 83%, much higher than in other age groups. Given concerns regarding availability of paid sick leave and perceived ability to comply with community mitigation strategies (e.g., self-isolation, social distancing, school dismissal) vaccination is becoming an increasingly critical part of the public health strategy aimed at reducing morbidity and mortality due to pandemic H1N1.

Only 3 target groups could be specifically examined using the data collected. Since children less than 6 months of age are at high risk of influenza-related complications and cannot be vaccinated, it is recommended that their caregivers receive the H1N1 vaccine. In this study, 80% (4/5) of respondents with infants in the home reported an intention to be vaccinated. Similarly, since many cases have occurred in school and day care settings and among healthy young adults, children between 6 months and 18 years old and young adults between 19 and 24 years of age are also encouraged to receive the vaccine. However, parents reported lower intention to vaccinate their older children and fewer young adults reported an intention to be vaccinated as recommended.

At this time of this study, self-isolation at home for a period of 7–10 days after symptom onset or 24 hours after resolution of fever (whichever is longer) was recommended by the North Carolina Division of Public Health for all persons with influenza-like illness. Nearly half of all families with children under age 18 living at home indicated that they could keep children at home, away from school or day care for at least 7–10 days. However, by the time of the survey's implementation, school dismissals, which were prevalent in the spring of 2009, were being recommended only for schools where all or most students were at high risk [16]. Similarly, 65% of those who worked part- or full-time indicated that they could stay home, away from work, for this amount of time. Those who are self-employed may be able to miss work for this period of time without sanction from their employer; however, they may forego income during this time. Legislative efforts to provide 5 days of sick leave benefits to all U.S. workers with H1N1 pandemic influenza have been unsuccessful to date [17].

Reasons cited for not intending to get vaccinated included not believing themselves to be at risk for infection, concern about side effects of the vaccine, and the belief that illness caused by pandemic H1N1 was likely to be mild. These same reservations have been expressed in studies conducted in Canada and Australia that examined attitudes toward pandemic vaccines and pandemic influenza [18], [19].

Importantly, most information received about pandemic H1N1 is from television sources. While the target groups for the pandemic H1N1 vaccine are different than those for seasonal influenza vaccine, this population-based survey indicates intention to receive pandemic vaccine is similar for adults in all age groups and for households with children. Since a relatively small number of respondents (n = 23) indicated that they were not included in a target group for the H1N1 vaccine, it seems likely that respondents were unaware of the target groups for this new vaccine. Similarly, only about one-third knew that two doses of vaccine may be recommended, suggesting that specific information regarding the vaccine had not been widely disseminated by the television media by the time of the assessment.

This study had several limitations. The study was cross-sectional and can only provide a picture of the population at a given point in time. This may be particularly important in this case, as pandemic H1N1 has been covered extensively in the media and attitudes and awareness may be affected with the amount of attention. The media attention surrounding the H1N1 pandemic is much more extensive than that seen during a usual influenza season. Also, due to the nature and the timing of the survey, respondents were asked to report their intention to receive both pandemic and seasonal vaccines. Reported intentions cannot be confirmed, and results may have been different if the pandemic vaccine had been available at the time of the survey. Finally, while these results are generalizable to the 2 county study area, no conclusions can be drawn about the intention to receive vaccine among the total population of North Carolina or any other group.

While the sample size of this study is small, the rapid needs assessment methodology is designed to be representative of the source population and has been widely validated [9], [20], [21], [22], [23]. However, if those who are most likely to be suspect of or refuse vaccination are also more likely to be missed in this survey (e.g., because they did not answer the door or declined to participate in the survey) there is potential for response bias. To minimize this problem, interviews were conducted on weekends and weekdays during both day and early evening hours and interviewers identified themselves as working with both the university and the health department.

As this pandemic unfolds, public health practitioners are grappling with many additional questions related to vaccine implementation and much remains for further study. Future research should examine pandemic H1N1 vaccine uptake in the population, particularly among target groups. Although longitudinal follow-up of the same individuals before and after vaccine implementation would be ideal, the rapid assessment methodology provides a representative sample of the study population, so future studies could use similar methodology for valid comparison using cross-sectional data. Additional studies should measure success of public health messaging related to pandemic H1N1 vaccine target groups, vaccine safety, and vaccine distribution. Lessons learned from public messaging around H1N1 may be applied to other large-scale public health events, as well as to annual seasonal influenza communication. Investigating these issues will help with effectively and efficiently preparing for and responding to the next influenza pandemic.

## Supporting Information

Appendix S1 (0.04 MB DOC)
