# Supplementary material for: Intent to Receive Pandemic Influenza A (H1N1) Vaccine, Compliance with Social Distancing and Sources of Information in NC, 2009
Source: PLoS One. 2010 Jun 18;5(6):e11226. doi: 10.1371/journal.pone.0011226 (PMC2887902; doi:10.1371/journal.pone.0011226)
Supplement: Appendix S1 — (0.04 MB DOC) [file pone.0011226.s001.doc]

APPENDIX S1.Survey instrument used to collect data related to intention to receive pandemic H1N1 and seasonal influenza vaccination, North Carolina, 2009 (N=207).

**Introduction and Request to Participate**

Are you willing to participate in this survey?

YES

NO

1. Gender: [INTERVIEWER RECORDS]

FEMALE

MALE

**Knowledge**

2. To the best of your knowledge, is there currently a vaccine being prepared for the pandemic influenza strain referred to as H1N1 or swine flu?

YES

NO

DON’T KNOW

3. Were you aware that 2 separate doses of an H1N1 vaccine are recommended?

YES

NO

DON’T KNOW

**Perceived Risk**

4. How concerned are you that you or someone in your household will be infected with H1N1 swine flu this fall or winter?

VERY CONCERNED

SOMEWHAT CONCERNED

NOT AT ALL CONCERNED

**H1N1 Vaccine**

5. Do you intend to get vaccinated against H1N1 swine flu when the vaccine is available later this fall?

YES

NO

DON’T KNOW

IF NO TO Q5, THEN Q 6, IF YES, THEN Q 7 AND Q 8.

6. If NO:

I’m going to read a list of possible reasons why someone might choose not to get the H1N1 swine flu vaccine. Please tell me if each of the following is a reason that you do not intend to get vaccinated. Y/N for each option below.

1. I do not think I will be infected with H1N1 swine flu [Y/N]

2. I do not think H1N1 swine flu will cause serious illness even if I am infected [Y/N]

3. I do not think the vaccine will prevent infection [Y/N]

4. It’s inconvenient to take a vaccine that requires 2 doses [Y/N]

5. I’m worried about possible side effects [Y/N]

6. I’m worried about taking a vaccine that may contain thimerosal [Y/N]

7. I’m not a member of a target group to receive the vaccine, such as a health care worker, young adult under age 24, or a pregnant woman. [Y/N]

8. It costs too much [Y/N]

9. I do not know where to get vaccinated [Y/N]

10. I do not have transportation to get vaccinated [Y/N]

11. I have immunity because I was already infected with H1N1 [Y/N]

12. I do not have health insurance [Y/N]

88. Other [Y/N] [OTHER TEXT]

7. If YES:

Please tell me if each of the following is a reason that you intend to get vaccinated against H1N1 swine flu. Y/N for each option below.

1. I’m worried about getting sick [Y/N]

2. I’m a member of a target group recommended to receive the vaccine, such as a health care worker, young adult under age 24, or a pregnant woman. [Y/N]

3. The vaccine was recommended by my health care provider [Y/N]

4. I want to keep others in my household from getting sick [Y/N]

88. Other [Y/N] [OTHER TEXT]

8. IF YES:

Where do you intend to get the H1N1 swine flu vaccine? [INTERVIEW READS CHOICES]

1. Private health care provider

2. Local health department

3. Hospital

4. Community health clinic

5. Employer

6. College or university health services

7. Pharmacy

88. Other [OTHER TEXT]

**Seasonal Influenza Vaccine -** ALL ANSWER Q 9

Now we’re going to ask a few questions about *seasonal influenza, the kind that occurs each year in the late fall and winter here in the United States*.

9. Did you receive a *seasonal* flu vaccine for the 2008-2009 flu season (last year)?

YES

NO

DON’T KNOW

IF NO, THEN Q 10. IF YES, THEN Q 11.

10. IF NO:

What was the primary reason you did not receive the *seasonal* flu vaccine for the 2008-2009 flu season? [SELECT ONE PRIMARY REASON]

1. I did not think I would be infected with flu

2. I did not think the flu will cause serious illness even if I was infected

3. I did not think the vaccine would prevent infection

4. I was worried about possible side effects

5. I was worried about taking a vaccine that contained thimerosal

6. I was not a member of target group such as a health care worker, someone with a chronic disease, or someone over 50 years of age

7. It cost too much

8. I did not know where to get vaccinated

9. I did not have transportation to get vaccinated

10. I had immunity because I was already infected with seasonal flu

11. I did not have health insurance

88. Other [OTHER TEXT]

REMINDER: ALL ANSWER Q 11.

11. Do you intend to get a *seasonal* flu vaccine for the 2009-2010 flu season (this year)?

YES

NO

DON’T KNOW

IF NO, THEN Q 12 . IF YES, THEN Q 13. ALL ANSWER Q 13.

12. IF NO:

I’m going to read a list of possible reasons why someone might choose not to get the seasonal flu vaccine. Please tell me if each of the following is a reason that you do not intend to get vaccinated for seasonal flu this year. [Y/N for each]

1. I think it’s more important to get the H1N1 swine flu vaccine and I don’t want to get both [Y/N]

2. I do not think I will be infected with seasonal flu [Y/N]

3. I do not think seasonal flu will cause serious illness even if I am infected [Y/N]

4. I do not think the vaccine will prevent infection [Y/N]

5. I’m worried about possible side effects [Y/N]

6. I’m not a member of target group such as a health care worker, someone with a chronic disease, or someone over 50 years of age [Y/N]

7. It costs too much [Y/N]

8. I do not know where to get vaccinated. [Y/N]

9. I do not have transportation to get vaccinated. [Y/N]

10. I have immunity because I was already infected with seasonal flu [Y/N]

11. I do not have health insurance [Y/N]

88. Other [Y/N] [OTHER TEXT]

REMINDER: ALL ANSWER Q13

13. Have you ever received a seasonal influenza vaccine?

YES

NO

DON’T KNOW

**Isolation and Social Distancing**

14. What is your current employment status?

1. WORK FULL TIME FOR PAY

2. WORK PART TIME FOR PAY

3. HOMEMAKER

4. STUDENT

5. RETIRED OR DISABLED

6. CURRENTLY UNEMPLOYED

88. OTHER

IF Q 14 = 3, 4, 5, 6, OR 88, THEN SKIP TO Q16. OTHERWISE ANSWER Q15.

15. IF EMPLOYED (IF Q 14 = 1 OR = 2 (FULL-TIME OR PART-TIME)):

Do you have any paid sick leave?

YES

NO

DON’T KNOW

16. Suppose you had H1N1 swine flu and health officials recommended that you stay at home, away from people who were not part of your household. For how many days would you be able to stay home?

1. 0 DAYS

2. 1-3 DAYS

3. 4-6 DAYS

4. 7-10 DAYS

88. OTHER [OTHER TEXT]

99. DON’T KNOW

17. Do you have any children age 18 or under living at home?

YES

NO

IF NO, THEN SKIP TO Q 22.

18. IF YES:

Do you have children who are [Y/N for each]:

1. Less than 6 months old [Y/N]

2. Between 6 months and 4 years old [Y/N]

3. Between 5 years and 18 years old [Y/N]

19. Do you intend to vaccinate your child(ren) for H1N1 flu?

YES

NO

DON’T KNOW

20. Are any of the children enrolled in school or daycare?

YES

NO

21. If your child had H1N1, what is the longest period of time that you would be able to keep your child at home, away from people who were not part of your household, without encountering major difficulties?

1. 0 DAYS

2. 1-3 DAYS

3. 4-6 DAYS

4. 7-10 DAYS

5. MORE THAN 10 DAYS

88. OTHER [OTHER TEXT]

99. DON’T KNOW

**Communications**

22. Before this interview, were you aware that H1N1 swine influenza is currently circulating in the community?

YES

NO

DON’T KNOW

23. Did you get information about H1N1 swine flu vaccine from any of the following sources? Y/N for each.

1. A health care provider [Y/N]

2. Employer or a co-worker [Y/N]

3. Family member or friend [Y/N]

4. Television [Y/N]

5. Radio [Y/N]

6. Newspaper (printed) [Y/N]

7. Internet [Y/N]

8. Centers for Disease Control and Prevention [Y/N]

9. State or local health department [Y/N]

88. Other [Y/N] [OTHER TEXT]

**Demographics**

24. What kind of health insurance do you have?

1. PRIVATE INSURANCE

2. MEDICARE

3. MEDICAID

4. I DON’T HAVE HEALTH INSURANCE

88. OTHER [OTHER TEXT]

99. DON’T KNOW

25. What is your age? [ENTER NUMBER]

26. What do you consider to be your race or ethnicity?

1. BLACK OR AFRICAN AMERICAN, NON-HISPANIC

2. WHITE, NON HISPANIC

3. HISPANIC

4. AMERICAN INDIAN OR ALASKA NATIVE

5. ASIAN

6. NATIVE HAWAIIAN OR PACIFIC ISLANDER

88. OTHER [OTHER TEXT]

**Thank You**
